# β-Caryophyllene-Loaded Microemulsion-Based Topical Hydrogel: A Promising Carrier to Enhance the Analgesic and Anti-Inflammatory Outcomes

**DOI:** 10.3390/gels9080634

**Published:** 2023-08-07

**Authors:** Sitah Alharthi, Zyta Maria Ziora, Gulam Mustafa, Pramila Chaubey, Ahmed Farag El Kirdasy, Ghallab Alotaibi

**Affiliations:** 1 Department of Pharmaceutical Sciences, College of Pharmacy, Shaqra University, Al-Dawadmi Campus, Al-Dawadmi 11961, Saudi Arabia; s_alharthi@su.edu.sa (S.A.); gulam@su.edu.sa (G.M.); cpramil@su.edu.sa (P.C.); 2 Institute for Molecular Bioscience, University of Queensland, St. Lucia, QL 4072, Australia; z.ziora@imb.uq.edu.au; 3 Department of Biochemistry, College of Veterinary Medicine, Sadat City University, Sadat City 32897, Egypt; ahmed.elkerdasy@vet.usc.edu.eg

**Keywords:** analgesia, nanocarrier, skin permeation, safety, bioavailability, microemulsion, dermatokinetics

## Abstract

Musculoskeletal pain and inflammation can vary from localised pain like pain in the shoulders and neck to widespread pain like fibromyalgia, and as per estimates, around 90% of humans have experienced such pain. Oral non-steroidal anti-inflammatory drugs (NSAIDs) are frequently prescribed for such conditions but are associated with concerns like gastric irritation and bleeding. In the present study, a microemulsion-based gel comprising β-caryophyllene, isopropyl myristate, Tween 80, and normal saline was prepared as a topical option for managing topical pain and inflammation. The globules of the microemulsion were below 100 nm with a zetapotential of around −10 mV. The drug entrapment was >87% with a drug loading of >23%. The permeation studies established better skin permeation (20.11 ± 0.96 μg cm^−2^ h^−1^) and retention of the drug (4.96 ± 0.02%) from the developed system vis-à-vis the conventional product (9.73 ± 0.35 μg cm^−2^ h^−1^; 1.03 ± 0.01%). The dermatokinetic studies established the better pharmacokinetic profile of the bioactive in the epidermis and dermis layers of the skin. The anti-inflammatory potential in carrageenan-induced rat paw oedema was more pronounced than the conventional product (~91% vis-à-vis ~77%), indicating a better pharmacodynamic outcome from the developed system. The nanotechnology-based natural bioactive product with improved efficacy and drug loading can provide a better alternative for the management of musculoskeletal pain.

## 1. Introduction

Microemulsions are emulsions produced in nanometer sizes to enhance the delivery of pharmacologically active substances. These are the thermodynamically stable isotropic systems in which an emulsifying agent, like surfactant and co-surfactant, combines two immiscible liquids into a single dispersed phase. Microemulsions typically have droplet sizes generally less than 200 nm. The size and shape of the particles scattered in the continuous phase is the primary distinction between an emulsion and a microemulsion. These carriers are solid spheres with an amorphous, lipophilic, negative-charged surface. Oil, emulsifying agents, and aqueous phases are the three primary elements of such systems [1,2,3].

With the advent of nanotechnology, especially the concepts of drug delivery, numerous formulations are available in the market with better safety and efficacy than conventional products. Using novel methods and materials has resulted in promising supramolecular structures that create a conducive environment for better delivery, especially by the topical route [4,5,6,7]. Though most of the marketed formulations are liposomal, a number of products based on microemulsions are also available for the benefit of humanity. Examples of such marketed products are the emulsified products of propofol, dexamethasone, flurbiprofen, and fat-soluble vitamins [8].

The natural bicyclic sesquiterpene β-caryophyllene (trans-(1,9)-8-methylene-4,11,11-trimethylbicycloundec-4-ene) is present in many plants and essential oils, including the oil from *Copaifera langsdorffii* (*Leguminosae*). Owing to its analgesic, antioxidant, antibacterial, and anti-inflammatory properties, β-caryophyllene is being explored as a bioactive molecule and in the form of novel drug delivery systems. Though β-caryophyllene is a promising molecule, challenges like volatility, poor aqueous solubility, and poor oral bioavailability provide scope for further nanotechnological research. The therapeutic ability of *C. langsdorffii* oil resin in concentration ranges of 1–10% has been employed by various researchers. To examine the potential by the topical route 1%, β-caryophyllene has also been explored. In the recent past, multiple nanointerventions like self-emulsifying systems and hydrogels for the delivery of this bioactive have been explored, though phospholipid-based microemulsions have not been prepared for topical delivery [9,10,11].

Microemulsions have proved their safety and efficacy in improving the performance of various drugs and bioactives. Being thermodynamically stable sub-micron emulsions, the drug-loaded globules find easy access to the biological membranes. Some researchers also use the term microemulsion for nano-range emulsions, though these are thermodynamically stable [12,13]. If phospholipid is one of the components, then biocompatibility and bio-integration are the added benefits [5,14]. Henceforth, in an attempt to explore the effect of the nanocarrier on the overall performance of the drug, without the use of ethanol, it was envisioned to formulate a microemulsion-based β-caryophyllene-loaded novel formulation. The approach of this research work is to provide targeted delivery of β-caryophyllene as well as modulation of the drug release rate to the target site, i.e., the region of nociceptors (epidermis and dermis) [15].

Isopropyl myristate (IPM) was used as oil due to its consistency (neither too light nor too viscous) and penetration enhancement effects. Natural oils are prone to oxidation, and batch-to-batch variability is observed in those oils [16]. Tween 80 was used as the surfactant based on exhaustive literature reports [12,17]. Most of the time, ethanol has been used as one of the components of microemulsions, but it is reported to be an irritant. To maintain the skin penetration characteristics and phospholipid dissolving attributes, propylene glycol was employed. Phospholipid has been incorporated due to the obvious reasons of biocompatibility, skin permeation, and drug retention properties [3,18]. In the present studies, dermatokinetic investigations were also performed to understand the pharmacokinetic profile of β-caryophyllene in the dermis and epidermis. The composition, carrier system, and approach for the topical delivery of β-caryophyllene in the conditions of pain and inflammation are the merits of the present study.

## 2. Results and Discussion

### 2.1. Pseudo-Ternary Phase Diagram

**Figure 1:**
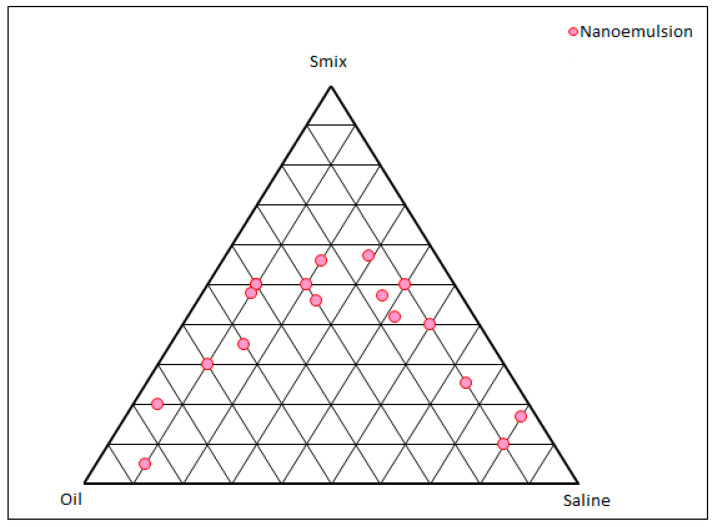
Pseudo-ternary phase diagram constructed between IPM, Smix, and normal saline. Each vertex of the triangle shows 100% of the mentioned component and every circular point shows the boundary between the monophasic and biphasic region.

The pseudo-ternary phase diagram obtained from IPM, the Smix, and normal saline are shown in Figure 1. The pink dots show the boundary of the monophasic region, beyond which the compositions will not result in any microemulsion dispersion. As reported by various research groups, polysorbate 80 can emulsify substantial amounts of oil, though the other two components, viz., phospholipid and propylene glycol, also play a vital role [17]. The ternary phase diagram has fetched the complete region where a formulator can explore numerous compositions that can form microemulsions. It also provides the boundary for which afterwards there are no chances of microemulsion formation. From Figure 1, a total of three compositions of the microemulsions were explored, containing almost the same oil composition as the drug is lipophilic. An ideal condition of drug to oil ratio, i.e., 1:5, was maintained in this study. Higher oil ratios will result in unnecessary cost escalation and lower percentages can result in lower drug entrapment and loading. The compositions of the three compositions are shown in Table 1.

### 2.2. Characterisation of the Microemulsions

The results obtained from the various characterisation studies have been presented in Table 2 for easy comparison and understanding. Practically, all the developed compositions were transparent as vivid from the optical observation as well the % transmittance values. All the values of % transmittance were >92% advocating substantial optical clarity. The values of % transmittance were of non-significant difference for the formulations ME1 and ME2, whereas the formulation ME3 showed a significant difference owing to a higher percentage of Smix (*p* < 0.05). The refractive indices of the developed systems ranged between 1.39 and 1.46, with that of ME1 and ME2 being almost similar. However, the refractive index of the formulation ME3 was significantly higher than both (*p* < 0.05), plausibly due to the higher percentage of polysorbate. The refractive index of water, Tween 80, and IPM are 1.33, 1.47, and 1.43, respectively. The values of the developed systems ranged between 1.33 and 1.47 and were in accordance with the published reports [19]. The conductivity values of all the developed nanoformulations were statistically different from each other (*p* < 0.05), ranging from 19.09 to 43.18 µS. The composition ME2 contained the highest amount of normal saline; hence, the conductivity was of the highest magnitude for it, whereas its value was least for ME1 reflected the least conductivity value. The pH values of all the compositions were well within the acceptable range of topical products. The normal pH value of the skin is around 5, and the skin can withstand pH up to 10.5 without showing any irritation. These physicochemical parameters of the microemulsions are crucial as substantial changes in these indicate microscopic structural changes in such systems [20].

The globule size of the developed nanosystems ranged from 79.18 nm to 95.07 nm in concordance with the % transmittance values. The most optically clear system offered the least size and vice-versa. The composition ME3 encompassed the maximum level of surfactants and offered the least globule size, as expected. However, the size range of all the developed formulations was below 100 nm, as desired from microemulsions [21]. The PDI values of the developed systems were well below the boundary of 0.3, assuring the reliability of the average globule size [22]. Being microemulsions derived from nonionic surfactants, the zeta-potential values of all the formulations were almost zero, though the systems were relatively stable. The nonionic surfactants stabilise the system by steric hindrance of the hydration layer on the hydrophilic headgroups rather than the electrostatic repulsion [23]. Pharmacokinetically, nanocarriers with zeta-potential values ≤± 10 mV are regarded as neutral supramolecular assemblies in biological systems [7]. In both EE and DL, it was observed that the compositions offered decreasing values with increasing Smix ratios for almost the same amount of oil and drug. Formulation ME2, having intermediate levels of surfactants, exhibited the highest EE as well as DL vis-à-vis ME2 and ME1 (*p* < 0.05). A possible explanation for such behaviour is that a higher concentration of surfactants might have extracted the drug molecules from the IPM globules into the dispersion medium, decreasing the β-caryophylline amounts in the globules. Literature reports also provide inferences for the dependence of entrapment efficiency on the type and amount of the surfactant(s) [24]. Based on the entrapment efficiency and drug loading capacity, the formulation ME2 was selected for further studies. Its globule size was also in the desirable range and it utilised moderate amounts of surfactants.

### 2.3. Transmission Electron Microscopy

**Figure 2:**
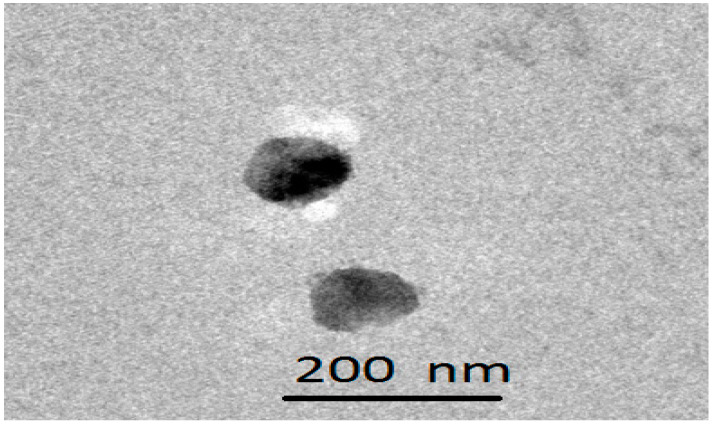
Transmission electron microphotograph of a frame of the selected microemulsion, i.e., ME2.

The microphotograph obtained from TEM is shown in Figure 2. Electron microscopy, an indispensable characterisation technique for microemulsions, can decipher vital information about nanocarriers. The microscopic vision of the nanoconstructs provides an idea about the topography and aggregation [25]. From the figure, it is clear that spherically shaped globules smaller than 200 nm have formed in the system. The surface of the globules appears to be smooth, and there is no evidence of any aggregation. The findings are in consonance with the previously published reports [26].

### 2.4. Gel Attributes and Rheology

**Figure 3:**
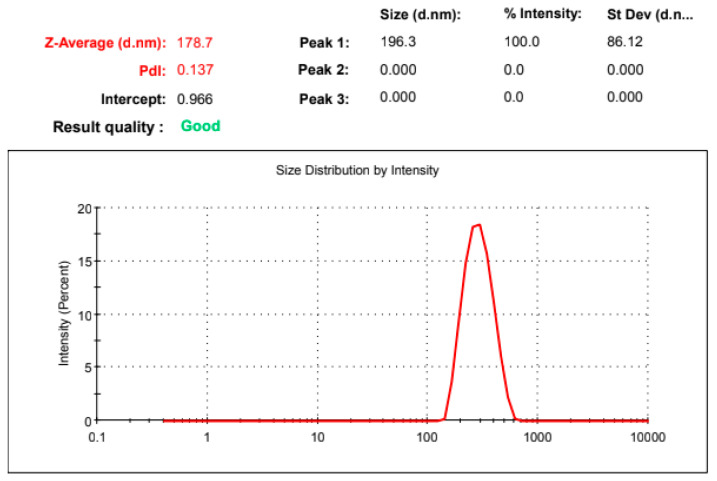
Particle size distribution of the developed microemulsion gel.

The gel fraction of the developed system was 95.19 ± 5.32%, and the sol fraction was 4.82 ± 0.21%, indicating a higher gel fraction. The swelling index of the gel was found to be 158.32 ± 12.39%. The obtained results were in consonance with the published reports [27]. The particle size of the gel was observed to be 178.7 nm, as shown in Figure 3.

**Figure 4:**
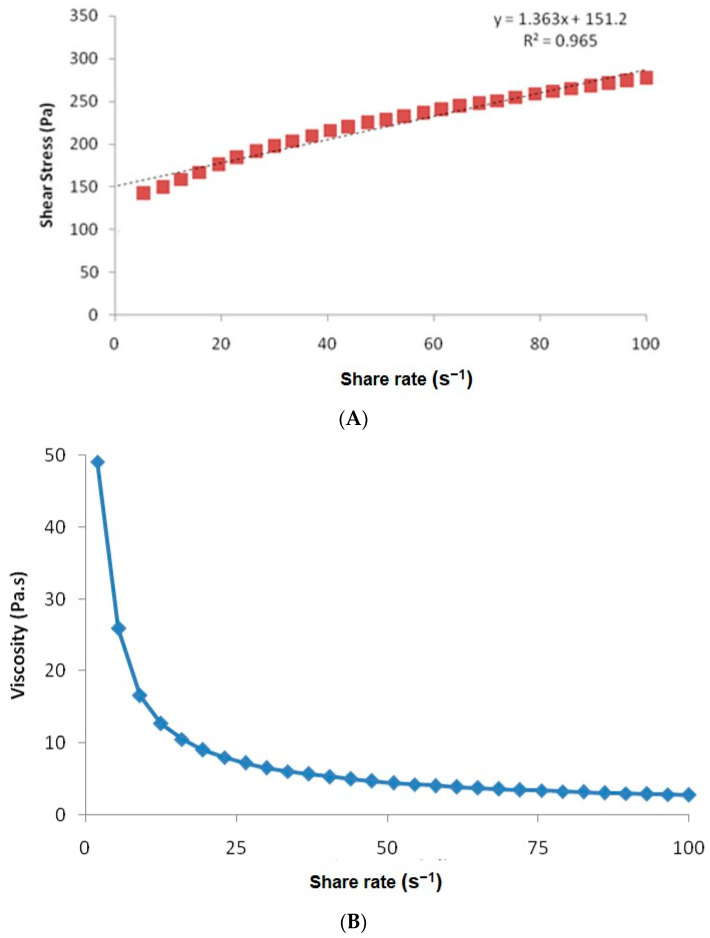
(**A**) Rheogram of the hydrogel prepared from the selected microemulsion. (**B**) The variation of viscosity as a function of shear rate.

The rheogram of the developed nanogel is shown in Figure 4A, and the graph between the shear rate and viscosity is depicted in Figure 4B. The graph shows that the shear rate and shear stress show a non-linear relationship, representing the characteristics of a typical non-Newtonian system with a yield value of 151.2 Pa. The developed hydrogel offered an average viscosity of 7.74 Pa·s or 7740 cP, though the initial viscosity was quite high with 49.1 Pa·s at the shear rate of 2 s^−1^, which decreased gradually with an increase in the shear rate. It is a characteristic feature of the shear thinning system, as is the case for the carbopol gels. This viscosity is sufficient enough for easy application and adherence, as per the published reports [28]. It will not allow the leakage of the gel from the container orifice.

**Figure 5:**
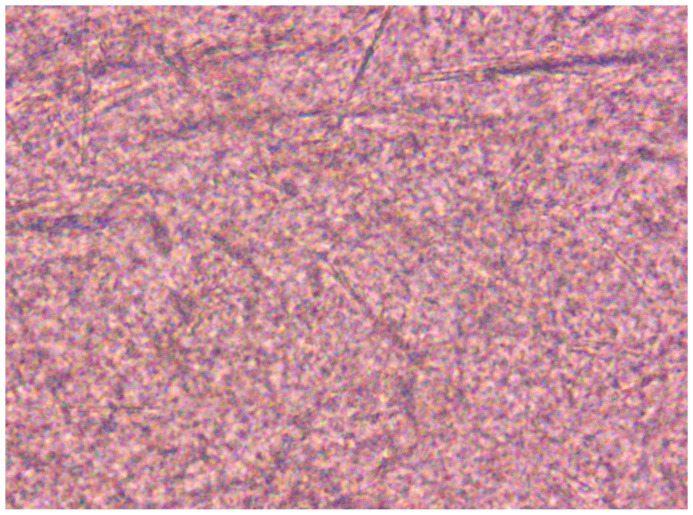
Optical microscopy of the developed gel at 1000×.

For internal quality control standards, optical microscopy of the developed gel was also performed at 1000×, as shown in Figure 5. The microscopic view was devoid of any un-swollen gel and particles.

### 2.5. Skin Permeation and Skin Retention

**Figure 6:**
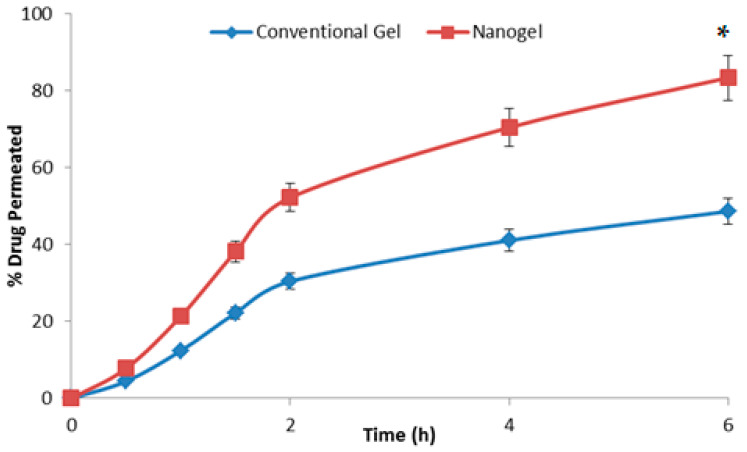
The graph showing the percent of β-caryophylline permeating across the rodent skin from the tested formulations with respect to time (*n* = 3). * indicates the significant differences at all the studied time points at *p* < 0.05.

The results of drug permeation across the skin are shown in Figure 6. It is clear from the graph that at every time point the amount of drug permeated across the rodent skin from the developed nanogel was substantially higher than that from the conventional gel (*p* < 0.05). The permeation studies were performed for 6 h since topical products, especially gels, are not retained on the body surface beyond this timeframe. In 6 h, the % of drug permeated from the nanogel was 83.42 ± 4.71%, and the value from the conventional gel was 48.66 ± 3.59%, almost 1.7 times lower than the novel formulation. The average drug permeation flux for the nano hydrogel was calculated to be 20.11 ± 0.96 µg cm^−2^ h^−1^, whereas the value for the conventional product was 9.73 ± 0.35 µg cm^−2^ h^−1^ (*p* < 0.05). The results clearly indicated that the developed system can deliver β-caryophylline at a relatively improved rate and in higher amounts across the skin layers. The composition as well the size of the nanoglobules of the microemulsion have resulted in improved delivery [5]. The formula for the calculation of the average permeation flux is shown below:

$$\text{Average permeation flux}=\frac{\text{Total amount of drug permeated}}{\text{Area of skin}\times \text{Time of permeation study}}$$

The drug retention values for the conventional gel and the developed microemulsion gel were observed to be 1.03 ± 0.01% and 4.96 ± 0.02%, respectively. The developed nano hydrogel was able to retain a substantial amount of drug within the skin layers (*p* < 0.5), ensuring a depot formation for durations beyond the 6 h of application [29].

### 2.6. Dermatokinetics

**Figure 7:**
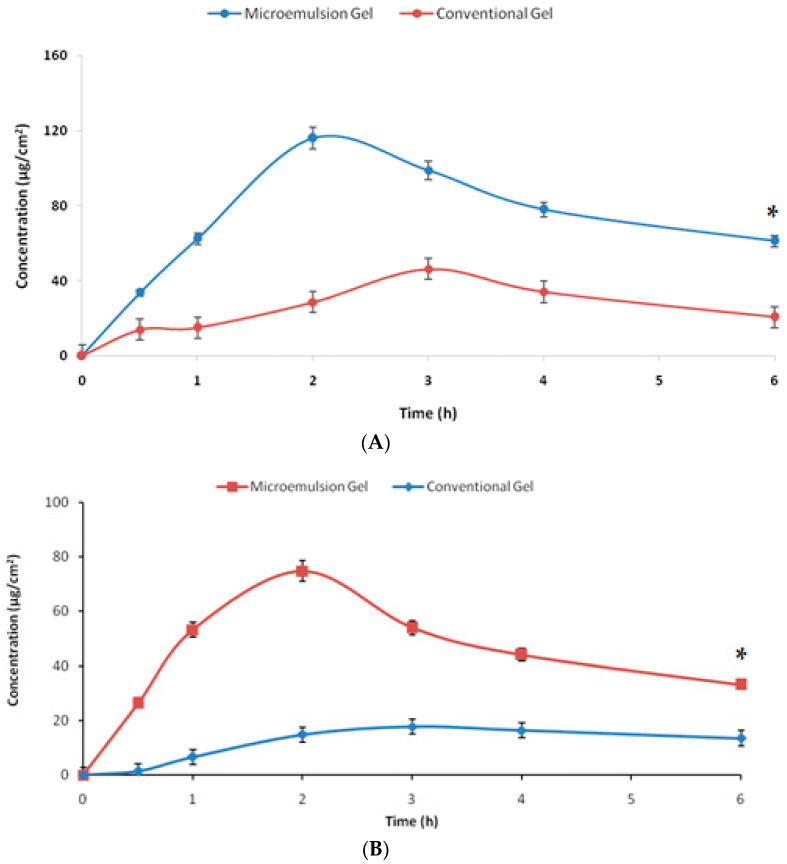
The graph showing the amount of β-caryophylline per unit area permeating across the (**A**) epidermis and (**B**) dermis from the tested formulations with respect to time (*n* = 3). * indicates the significant differences at all the studied time points at *p* < 0.05.

The results of the dermatokinetic studies are shown in Figure 7. The amount of drug per unit of skin area was higher for the developed system at every time point with respect to the conventional gel (*p* < 0.05). The penetration offered by the microemulsion gel was substantially higher for the studies of the microemulsion gel as the Cmax achieved was faster vis-à-vis the conventional gel. The first-order permeation constant (K_p_) for the developed microemulsion gel across the epidermis was 4.01 h^−1^,and it was 1.19 h^−1^ for the conventional gel, while for the dermis the respective values were 3.21 h^−1^ and 0.96 h^−1^, signifying the substantially higher penetration potential, owing to the benefits of the components and microemulsion (*p* < 0.05). The values of Cmax obtained for the microemulsion gel and the conventional gel in the epidermis were 192.53 µg/cm^2^ and 114.51 µg/cm^2^, at the respective Tmax of 2 h and 3 h, indicating a faster delivery of the drug by the microemulsion-based hydrogel vis-à-vis the plain hydrogel. There was an almost 1.7-fold enhancement in the maximum drug delivered to the epidermis. Analogously, the Cmax achieved in the dermis was 138.80 µg/cm^2^ for the developed nanosystem at the Tmax of 2 h and 39.11 µg/cm^2^ for the conventional system at the Tmax of 3 h, signifying the delivery potential of the developed system. There was an almost 3.5-fold enhancement in the maximum drug delivered to the dermis. The topical bioavailability parameter, i.e., AUC, was 502.93 µg/cm^2^ h for the epidermis by the developed microemulsion gel vis-à-vis 168.67 µg/cm^2^ h for the conventional system indicating almost three times the enhanced potential of drug delivery by the developed system. In the dermis, the respective values were 758.35 µg/cm^2^ h and 408.50 µg/cm^2^ h by the novel system and the conventional gel. All the dermatokinetic parameters have been tabulated in Table 3. The enhancement of the topical bioavailability in the epidermis was almost three times and in the dermis it was almost twice, which is substantially higher. All the dermatokinetic parameters of the developed system were far superior than the conventional product, thus indicating huge potential of the developed nanocarrier for delivery of this bioactive by the topical route [26]. In pain conditions, the target nociceptors are located in the areas of the dermis and the epidermis, and the developed system significantly delivered the bioactive molecule at these sites, indicating huge potential in the management of such conditions as pain and inflammation [15].

### 2.7. Carrageenan-Induced Rat Paw Oedema

**Figure 8:**
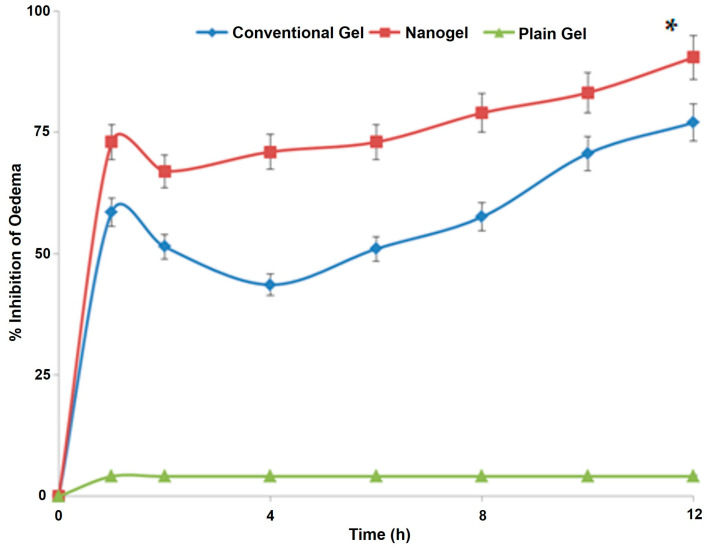
The graph showing variation of the % oedema in the paws of rodents receiving various treatments (*n* = 3). * indicates the significant differences at all the studied time points at *p* < 0.05.

The results obtained from the most widely used anti-inflammatory model have been reported in Figure 8. The group of animals (*n* = 3) receiving the plain gel (no drug) showed no substantial change in the paw oedema, indicating that the compositions employed in the microemulsion formulation, viz., IPM, phospholipid, propylene glycol, carbopol, and saline do not have any significant anti-inflammatory action of their own. However, both the conventional gel and the nano hydrogel substantially reduced oedema over a while (*p* < 0.05). However, the results obtained from the nano hydrogel were far superior to that from the conventional gel at every time point (*p* < 0.05). At the last time point of the study, i.e., 12 h, the group receiving the nano hydrogel was almost 91% relieved from the inflammation, whereas this anti-inflammatory value for the animals receiving the plain gel was around 77%. The enhanced efficacy of the developed system over the conventional product has been established using a reliable in vivo model. These studies demonstrated the potential of the designed carrier to deliver the natural bioactive in substantial amounts to induce its anti-inflammatory potential. The findings are unique and motivating as the desired effect was elicited in a shorter duration of time and was prolonged substantially.

## 3. Conclusions

The present study explored the possibility of a scalable β-caryophylline-loaded microemulsion-based hydrogel for topical applications in pain-related disorders. The formulation employed generally recognised as safe (GRAS) ingredients and simple formulation techniques and resulted in a product that can not only deliver the drug to the interiors of the skin but can also enhance its efficacy. The present strategically designed nanoformulation inherits huge scope and promise in treating musculoskeletal pain-related disorders by the topical route while avoiding unnecessary exposure of the body to the systemic concentration of drugs employed like NSAIDS in the management of pain-related disorders. The dermatokinetics studies have provided a clear indication that the pharmacologically active molecule was delivered in the various skin layers in substantial amounts and in an appreciable manner, vouching for the potential of such systems in delivering bioactives to pain target sites by the topical route.

## 4. Materials and Methods

### 4.1. Materials

β-caryophyllene, triethanolamine, and carrageenan were purchased from Sigma-Aldrich (St. Louis, MO, USA). Isopropyl myristate (IPM), Tween 80, phospholipid, buffer reagents, and dialysis membrane were procured from HiMedia (Maharashtra, India). Phospholipon 90 G (phospholipid) was procured from Lipid LLC, Ludwigshafen, Germany. Propylene glycol (1,2-Propanediol) was supplied by Merck Millipore, Mumbai, India. Carbopol 940 (carbomer) was supplied by Lubrizol India Pvt. Limited, Mumbai, India. In-house double distilled water was used throughout the studies.

### 4.2. Methods

#### 4.2.1. Preparation of Microemulsions

A pseudo-ternary phase diagram was prepared between the oil (IPM), surfactant mix (Smix; Tween 80, phospholipid, and propylene glycol in the mass ratio of 5:0.1:0.1), and normal saline. Other ratios were also tried, but phospholipid amounts greater than 2% resulted in a hazy Smix composition. The reasons for the component selection have already been disclosed in the introduction section. In brief, dilutions of oil and Smix were prepared in the mass ratios of 9:1 to 1:9 and titrated with normal saline. The separation of phases, appearing as haze or gelling, was the visual end point of the titration. Analogously, dilutions of Smix with saline were prepared and titrated with IPM until the end. All the percentage values of the three components for each titration were plotted on a ternary phase diagram to demarcate the homogeneous (microemulsion) [3] region from the heterogeneous phase [30,31]. Three compositions were selected out of the homogeneous phase of the pseudo-ternary diagram, accounting for almost the same amount of oil, but different Smix and saline amounts. The compositions were deciphered from the microemulsion region, and phospholipid was dissolved in propylene glycol and IPM with stirring at 500 rpm. β-caryophyllene (equivalent to 1% *w*/*w*) was dissolved in it. After a clear solution formed, a weighed amount of Tween 80 was added. The calculated amount of saline was added in aliquots to the mixture to fetch with the microemulsion. The exact compositions of the three microemulsions have been disclosed in the results and discussion section [3].

#### 4.2.2. Characterisation of the Microemulsions

The microemulsions were characterised for various attributes ranging from optical clarity, pH, refractive index, conductivity, globule size, zeta-potential, polydispersity index (PDI), drug loading, and drug entrapment. Based on the comparisons of these attributes, the formulation to be gelled was selected.

#### 4.2.3. Optical Clarity, Conductivity, pH, and Refractive Index

For optical clarity, a simple UV-visible spectrophotometric technique was employed using the % transmittance feature of the spectrophotometer (Cary 100; Agilent Technologies, Hong Kong). The developed systems were observed for % transmittance at 650 nm against water as a blank [3]. A conductivity meter (Seven Excellence S100; Mettler Toledo, Hong Kong) was used to test electrical conductivity. The recordings were made when the electrode was dipped into the microemulsions and equilibrium was reached. A minimum of three observations were recorded for each recording [2,32].

pH determination of the undiluted formulations was performed on a TDS digital pH meter. The refractive index of the developed systems was measured using an Abby’s refractometer against water as the reference fluid at the ambient temperature.

#### 4.2.4. Micromeritics and Zetapotential

The globule size, globule size distribution, and the PDI were determined in triplicate using a particle size analyser based on dynamic light scattering (Litesizer 500; Anton Paar, Graz, Austria). Being an emulsified system, the dilution prior to size determination was avoided. The PDI was also provided by the same equipment while determining the average globule size from the globule size distribution. The same equipment determined the zeta-potential values of the developed microemulsions using electrophoretic light scattering in triplicate.

#### 4.2.5. Drug Entrapment and Drug Loading

To determine the amount of drug entrapped within the IPM globules, drug entrapment and drug loading were [4] determined using the dialysis method [33,34]. In brief, 1 mL of the microemulsion was packed in a dialysis bag (12 kDa; 2.4 nm) and suspended in 20 mL of ethanol. The system was maintained at ambient temperature, and the sink conditions were maintained by stirring at 100 rpm for two hours. After the stirring, an aliquot of the diffusion medium was analysed for the unentrapped drug by reverse phase high-performance liquid chromatography, using a well-established method [35]. For the used method, the limit of quantification was 0.398 μg/mL and the limit of detection was 0.119 μg/mL with a coefficient of determination of 0.9976. The entrapment efficiency was reported as the amount of the bioactive present inside the globules out of the total drug added, whereas the drug loading was reported as the amount of entrapped drug per 100 parts of the microemulsion, as per the following equations:

$$\text{Drug entrapment efficiency}=\frac{\left(\text{Total Drug}-\text{Diffused Drug}\right)}{\text{Total Drug}}\times 100$$

$$\text{Percent drug loading}=\frac{\text{Entrapped Drug}}{\text{Total carrier to entrap the drug}}\times 100$$

#### 4.2.6. Transmission Electron Microscopy

Out of the three developed microemulsions, the transmission electron microscopy of the selected composition was performed using negative staining of 1% phosphotungstic acid. After being pipetted onto the carbon grids, the microemulsion was dyed with a solution of 1% phosphotungstic acid. The stained microemulsion was then observed and captured on a transmission electron microscope (TEM) using Analysis 3.0 Software and a Hitachi H-7000 TEM equipped with a Mega View II Digital Camera.

#### 4.2.7. Development of Microemulsion Gel and Gel Characteristics

The formulation was intended to be applied topically in the gel form. To develop a microemulsion-based gel, the selected microemulsion was incorporated into a secondary hydrogel. The microemulsion-based hydrogel was prepared using Carbopol 940. Precisely, carbomer, equivalent to 1% of the total drug-loaded microemulsion, was added into the microemulsion. It was mixed properly and refrigerated overnight. The next day, triethanolamine was added equalling 1.5 times of the carbomer, and the carbomer was neutralised to form gel [36]. Analogously, a plain gel comprising 1% β-caryophylline, saline, carbomer, and triethanolamine was prepared for comparison. The optical microscopy of the microemulsion gel was also performed as a routine characterisation study. The microemulsion gel was diluted to 100 times with distilled water assisted with bath sonication and analysed for particle size as disclosed in the micromeritics and zetapotential section [37].

The sol-gel fraction analysis was also performed as per the standard procedure by making hydrogel discs with radii between 1 and 2 mm. These discs were dried at room temperature and further placed in an oven maintained at 45 °C until a constant weight was achieved. The dried discs were boiled for four hours in water and again dried as discussed above. The weights after drying were measured. The sol and gel fractions were determined as per the following equations, where “x” designates the weight of dry gel before boiling and “y” represents the weight of dry gel after boiling [27]:

$$\%\;\mathrm{Fraction}\;\mathrm{of}\;\mathrm{sol}=\frac{\left(x-y\right)}{x}\times 100$$

% Fraction of gel = 1 − % of sol fraction

The swelling index of the gel was also performed by dipping the pre-weighed discs as discussed above in distilled water and taking them out and re-weighing after 3 h, after removing the adherent water by blotting paper. The percent swelling was determined using the following equation, where “a” stands for the weight of “swollen disc” and “b” represents the weight of “dry disc” [27]:

$$\%\;\mathrm{gel}\;\mathrm{swelling}=\frac{\left(a-b\right)}{b}\times 100$$

#### 4.2.8. Rheology

The rheology of the developed gel was studied using a cup and bob rheometer (Model MC1, PaarPhysica). Approximately 5 g of the gel was placed in the cup and was allowed to rest for around 5 min. The shear stress value was increased, automatically, linearly from 0.1 to 100 s^−1^. From the rheogram, the viscosity of the gel was determined [38].

#### 4.2.9. Skin Permeation and Skin Retention Studies

Healthy unisex Laca mice were used for the drug permeation studies across the rodent skin. The animals (*n* = 3) were sacrificed by cervical dislocation, and the skin was harvested. All animal-handling procedures, as well as sample collection and disposal, were carried out following the Institutional Animal Care and Use Committee (IACUC) rules, with monitoring provided by the faculty of Veterinary Medicine at the University of Sadat City, Egypt, bearing approval number VUSC-022-1-23. The depilated skin was placed in the donor compartment of a Franz diffusion cell, with the outer portion facing the upper side of the donor compartment. The area of the donor compartment was 3.14 cm^2^ (as per the specifications of the manufacturer, PermeGear Franz Diffusion Cell), and the volume of the receptor compartment was 30.0 mL. The receptor compartment was filled with phosphate-buffered saline solution (PBS) of pH 6.8 containing 10% ethanol. A 1:1 volume ratio of buffer and ethanol has been reported earlier, but the 10% *v*/*v* solution offered sufficient sink medium for the natural bioactive, henceforth, it was used [10]. Formulations (developed nano hydrogel and the conventional gel) were applied on the exposed portion of the skin containing around 200 mg of accurately weighed gel. At predetermined time-points, a sample of 1 mL was withdrawn from the receptor compartment and replenished with an equal volume of fresh medium. The samples were filtered and analysed by reverse phase high-performance liquid chromatography [35].

For skin retention studies, the skin from the donor compartment was removed after the permeation studies and washed with water thrice to remove the adhering formulation. The skin was chopped into small pieces and placed in 20 mL of ethanol for 12 h for complete extraction of the drug. The filtered solvent was processed and analysed as disclosed earlier [39].

#### 4.2.10. Dermatokinetics

The dermatokinetic profile of the developed hydrogel and the conventional hydrogel were examined using the well-established method reported earlier. The animal skin was excised and mounted on the Franz Diffusion Cells as in the skin permeation and retention studies. The difference lies in the sampling technique. At each sampling time point, the whole skin was removed, washed off the adhered drug/formulation, and separated into the dermis and the epidermis using lukewarm water. Both the skin layers were extracted in ethanol for the drug and the amount of the drug was determined using HPLC, as reported previously. The study was carried out for 6 h and the sampling was conducted at 0.5 h, 1 h, 1.5 h, 2 h, 4 h, and 6 h. The obtained drug concentrations in both the skin layers at the sampling time points were analysed using one compartment open body model. From the data analyses, the important dermatokinetic parameters like respective permeation constant (Kp), respective layer elimination rate constant (Ke), area under drug concentration and time curve (AUC), maximum concentration achieved in the respective skin layer (Cmax), and the time required to achieve the respective Cmax (Tmax) were computed and compared [40,41,42].

#### 4.2.11. Carrageenan-Induced Rat Paw Oedema

For this pharmacodynamics evaluation, healthy unisex Wistar rats were employed. A total of five groups were made comprised of three animals each. The right hind paws of the rats in all groups received an intra-plantar injection of 0.05 mL homogeneous suspension of 1% carrageenan in sterile normal saline to cause local inflammation. The paw volume was assessed immediately after carrageenan administration (i.e., time zero). To calculate the immersed paw volume in mL, the hind paw was submerged in the measurement cell from the hairline to the ankle. This was measured using a plethysmometer and the volume displacement method (UgoBasile, Gemonio, Italy). After waiting for thirty minutes, 200 mg of the formulation to be evaluated was lightly rubbed on the surface of the right hind paws with the index finger without any dressing. At 1, 2, 4, 6, 8, 10, and 12 h after carrageenan administration, paw volume was assessed. A % increase in paw volume since time zero was calculated. The anti-inflammatory response’s time-course was identified [16]. The following equations were employed:

$$\mathrm{Percent}\;\mathrm{swelling}\;(\mathrm{PS})=\frac{\left(V-V0\right)}{v0}\times 100$$

$$\mathrm{Percent}\;\mathrm{inhibition}\;\mathrm{of}\;\mathrm{oedema}=\frac{\left(1-PSt\right)}{PSc}\times 100$$

V is the paw volume at different time intervals, V0 is the initial paw volume at time 0, PSt is the percent swelling in the test group, and PSc is the percent swelling in the control group.

#### 4.2.12. Statistical Analysis

To reach a statistical decision, the mean values were compared with each other using Student’s *t*-test and the analysis was performed using SPSS statistical software (SPSS version 10.0.1, Chicago, IL, USA).

## Figures and Tables

**Table 1:** The compositions of the selected microemulsions from the ternary phase.

Formulation Code	β-caryophylline (mg)	IPM (g)	Tween 80 (g)	Phospholipid (mg)	Propylene Glycol (mg)	Normal saline (g)
ME1	200 (1%)	2 (10%)	9.23 (46.15%)	185 (0.925%)	185 (0.925%)	8.2 (41%)
ME2	200 (1%)	2 (10%)	7.30 (36.5%)	150 (0.750%)	150 (0.750%)	10.2 (51%)
ME3	200 (1%)	2.2 (10%)	13.27 (66.35)	265 (1.325%)	265 (1.325%)	3.8 (19%)

**Table 2:** The values of % transmittance, refractive index, pH, conductivity, size, PDI, zeta-potential, entrapment efficiency (EE), and drug loading (DL) of the developed microemulsions (* indicates the significant difference at *p* < 0.05).

Code	% Transmittance	Refractive Index	pH	Conductivity (µS)	Globule Size (nm)	PDI	Zeta Potential (mV)	% EE	% DL
ME1	95.97 ± 0.17	1.39 ± 0.04	6.23 ± 0.18	23.92 ± 0.97 *	83.46 ± 1.93 *	0.198 *	−7.94 ± 0.04 *	71.52 ± 1.98 *	15.77 ± 1.52 *
ME2	92.19 ± 0.21	1.38 ± 0.01	6.19 ± 0.13	43.18 ± 1.15 *	95.07 ± 0.15 *	0.225 *	−3.09 ± 0.02 *	87.33 ± 2.17 *	23.08 ± 1.14 *
ME3	98.73 ± 0.19 *	1.46 ± 0.03 *	6.31 ± 0.25	19.09 ± 0.11 *	79.18 ± 0.27 *	0.274 *	−0.18 ± 0.01 *	68.01 ± 2.39 *	12.98 ± 1.49 *

**Table 3:** The dermatokinetic parameters obtained from the studied topical formulations for the dermis and the epidermis.

Dermatokinetic Parameter	Conventional Gel		Microemulsion Gel	
	Epidermis	Dermis	Epidermis	Dermis
K_p_ (h^−1^)	1.19 ± 0.02	0.96 ± 0.01	4.01 ± 0.13	3.21 ± 0.09
C_max_ (µg/cm^2^)	114.51 ± 2.03	138.80 ± 1.74	192.53 ± 2.69	39.11 ± 0.95
T_max_ (h)	3	3	2	2
AUC (µg/cm^2^ h)	168.67 ± 9.33	408.50 ± 11.91	502.93 ± 24.67	758.35 ± 33.33

## Data Availability

Not applicable.
